# Design of Digital-Twin Human-Machine Interface Sensor with Intelligent Finger Gesture Recognition

**DOI:** 10.3390/s23073509

**Published:** 2023-03-27

**Authors:** Dong-Han Mo, Chuen-Lin Tien, Yu-Ling Yeh, Yi-Ru Guo, Chern-Sheng Lin, Chih-Chin Chen, Che-Ming Chang

**Affiliations:** 1 Ph.D. Program of Electrical and Communications Engineering, Feng Chia University, Taichung 40724, Taiwan; 2 Department of Electrical Engineering, Feng Chia University, Taichung 40724, Taiwan; 3 Department of Automatic Control Engineering, Feng Chia University, Taichung 40724, Taiwan; 4 Master’s Program of Department of Computer Science and Engineering, National Chung Hsing University, Taichung 40227, Taiwan; 5 Master’s Program of Biomedical Informatics and Biomedical Engineering, Feng Chia University, Taichung 40724, Taiwan

**Keywords:** digital-twin, digital-twin human-machine interface sensor (DT-HMIS), DT-Sensor, PLC, Industry 5.0, finger gesture, virtual manipulation, extension-limbs, computer vision, mechatronics

## Abstract

In this study, the design of a Digital-twin human-machine interface sensor (DT-HMIS) is proposed. This is a digital-twin sensor (DT-Sensor) that can meet the demands of human-machine automation collaboration in Industry 5.0. The DT-HMIS allows users/patients to add, modify, delete, query, and restore their previously memorized DT finger gesture mapping model and programmable logic controller (PLC) logic program, enabling the operation or access of the programmable controller input-output (I/O) interface and achieving the extended limb collaboration capability of users/patients. The system has two main functions: the first is gesture-encoded virtual manipulation, which indirectly accesses the PLC through the DT mapping model to complete control of electronic peripherals for extension-limbs ability by executing logic control program instructions. The second is gesture-based virtual manipulation to help non-verbal individuals create special verbal sentences through gesture commands to improve their expression ability. The design method uses primitive image processing and eight-way dual-bit signal processing algorithms to capture the movement of human finger gestures and convert them into digital signals. The system service maps control instructions by observing the digital signals of the DT-HMIS and drives motion control through mechatronics integration or speech synthesis feedback to express the operation requirements of inconvenient work or complex handheld physical tools. Based on the human-machine interface sensor of DT computer vision, it can reflect the user’s command status without the need for additional wearable devices and promote interaction with the virtual world. When used for patients, the system ensures that the user’s virtual control is mapped to physical device control, providing the convenience of independent operation while reducing caregiver fatigue. This study shows that the recognition accuracy can reach 99%, demonstrating practicality and application prospects. In future applications, users/patients can interact virtually with other peripheral devices through the DT-HMIS to meet their own interaction needs and promote industry progress.

## 1. Introduction

In many situations or environments, people often need to operate peripheral devices with their hands or express their wishes through language. However, in certain cases, people may not be able to directly touch physical hardware devices or verbally express themselves, such as those who work in noisy environments, those who are too far away, those who cannot speak, or those with mobility impairments. Despite this, there is still a need for people who cannot or have difficulty wearing controllers to operate physical peripheral devices or express their ideas through verbal language. In the development of Industry 5.0, digital-twinning is an important advancement that enables human-machine interaction and collaboration through digital-twinning technology [1]. Digital-twinning technology can record or monitor the behavior of physical objects through sensors and generate corresponding digital-twin (DT) models. These models can reflect the specific situations of physical objects, and people can further adjust or modify DT models using technologies such as artificial intelligence to make them more stable or accurate. Digital-twinning technology is a very useful method that can help people better understand and control or investigate the specific behavior and data of physical objects.

Introducing a machine-readable semantic reasoning framework [1] into industrial manufacturing management models unsafe states in the production process and constructs high-fidelity virtual DT production areas. This virtual area can simulate various unsafe states in the production area and generate a virtual dataset. Then, the virtual dataset is mixed with the real dataset and used to train and test the detection network for reasoning unsafe cases mapped to the ontology to protect people’s safety. In addition, there is also a digital-twinning method [2] in industrial welding that aims to improve the geometric quality of welding by combining the newly developed steady-state shrinkage of convex hull volume (SCV) method for non-standard welding simulation with collected geometric data for 3D scanning of parts. The method consists of an analysis cycle of data collection, virtual assembly, non-standard welding simulation, and variation analysis. The method focuses on improving the geometric quality of welding in manufacturing, where sheet metal parts and machine parts are connected by laser welding. Digital-twinning based on non-standard welding simulation is used to predict the results of the welding process and evaluate the performance of digital-twinning by comparing it with the actual welding results. Several different welding simulation methods, including traditional transient simulation and new SCV methods, were evaluated based on simulation speed and their ability to predict actual welding results.

Twin modeling has also been applied in the field of biology, where a twin model of cows is used to monitor and assess the survival conditions and physiological cycles of a herd [3]. This helps improve the care and conditions of the cows. Additionally, DT models have been used in urban agriculture planning [4], where a DT-generated decision support system is used with an Internet-of-Things (IoT) Aquaponics Unit prototype. The system analyzes the DT of the fish-vegetable symbiosis unit to compare and predict production variables. Sensors placed in the fish tank and growth bed monitor data such as water and ambient temperature, humidity, fish feeding events, and pH levels. Sensor readings are digitized through a data collection system and transferred to a microprocessor via WiFi. The aggregated data undergoes pre-processing and initial analysis, including “if-then” rules, formatting, and error checking, before being transferred for storage, more intensive processing, and modeling. However, in terms of network transmission security applications, DTs [5] can also be used for monitoring and managing industrial IoT devices. The platform is considered a central virtual control unit for both static and dynamic sensor data and continuously receives real-time updates from static devices through the connection of IoT sensors. The collected data can be visualized in a digital 3D model. The platform can also monitor and provide virtual data analytics to help optimize Industrial-Internet-of-Things (IIoT) devices. There are many good examples of DT applications, from industrial applications to healthcare and agricultural planning.

This work categorizes DT sensing technology into three different types. The first type is based on touch or handheld DT sensing systems [6], which implement sliding gestures on the screen [7,8]. For example, individual gender and age can be predicted and estimated through data prediction using a mobile device, or data from touch screen scrolling or zooming, finger length, curvature, click, drag, pinch, pressure gestures, and data from built-in sensors in mobile devices [9,10]. The second type is a wearable DT sensing system for industrial use that can replace the first type of touch-based system. It is a framework for human-robot interaction (HRI) based on industrial 4.0 robots used in a mixed team. The proposed framework can be used in real-time with industrial robots. The physical equipment consists of collaborative robots and MR head-mounted devices (MR-HMDs), while the digital components include the DT model of the robot, additional virtual objects, and a user interface (UII) that can identify and consider human gestures, sound, and motion [11]. The system operates by transmitting data through the network of cyber-physical systems (CPS) in the industrial environment to control the motion of robots wearing heavy equipment. Nevertheless, the first and second types of DT virtual control technology based on handheld or wearable devices have drawbacks, such as being bulky and being unable to redefine customized control peripheral instructions from the users. Additionally, they can only adjust or guide the robot’s trajectory on-site and cannot generate special instructions through digital encoding. Because it requires heavy equipment to be worn and hardware assistance, it is not the focus of this article. Therefore, we focus on developing the third type of DT sensing system, which is non-wearable and non-contact, with the functionality of the first and second types, providing the advantages of non-contact and non-wearable features. The benefit of doing this is that users/patients can simply use the characteristics of a single hand finger to translate various digital codes in camera images into specific gesture instructions, which can be mapped and matched to control peripheral devices. Using DT image sensing gesture control can replace the need to remotely touch the target, which is a good design direction.

Thus, this study aims to develop an economically efficient and easy-to-install Digital-twin human-machine interface sensor (DT-HMIS). Overall, the paper presents a novel DT-HMIS design that does not require contact or wearable devices. The goal is to solve the problem of lacking physical interaction with machines, improve human-machine interaction, and potentially benefit a wide range of users/patients. Based on cost savings and ease of installation and deployment, this study will use computer vision and algorithms to create a DT-HMIS, which can be individually updated by users/patients through the storage for mapping tables and programmable logic controllers (PLCs) to control operational instructions, to address the aforementioned problems of human-machine interaction and customizable control capabilities. Users/patients will benefit greatly from this. The design of this system has the following simple application areas. For example, people with mobility difficulties who cannot quickly pull or touch buttons from a distance and can’t call for help or seek help in noisy situations and people who can’t speak. It also can be used in industrial, commercial, biological breeding care, child cognitive and maturity assessment, and dementia evaluation in medical fields [12].

## 2. Conceptual Design

Algorithm and configuration for human activity sensing and finger gesture recognition devices [13,14,15,16]. There are two types of non-wearable gesture recognition methods: the first emphasizes rule-based approaches, using designer-defined feature rules to identify input images [17]; the other focuses on integrating multiple learning methods and using collected data samples to train classifiers for gesture recognition, which requires high computational resources. In this study, the first method will be used to reflect finger features for finger gestures [18], generating double-digit value pairs for control combinations. This method is beneficial in saving computational resources, simplifying and reducing costs, and making hardware easily accessible to everyone, which is conducive to popularization.

**Figure 1:**
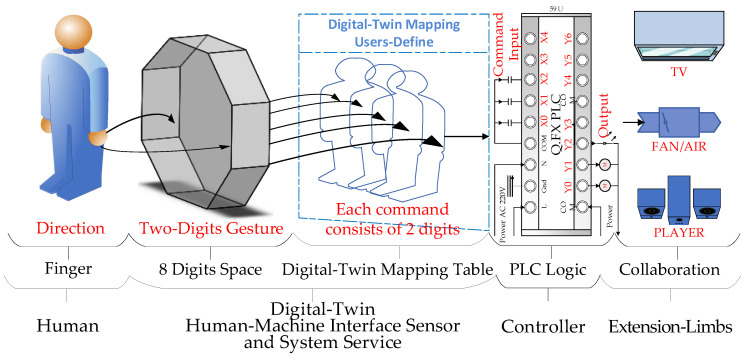
The process of transforming DT-HMIS into instruction control commands or reassembling speech.

The functionality and module architecture of the DT-HMIS is shown in Figure 1. When the camera captures finger gesture features, the machine vision algorithm in the image space divides the image into eight regions and assigns a single-digit number to each position, such as “1, 2, 3, 4, 5, 6, 7, 8”. The system then encodes the value in each region into a double-digit instruction. The double-digit instruction is referenced to the user/patient’s pre-defined digit twin model mapping table, which maps to the signal input point X of the controller. Then the user/patient can perform logical control unit programming based on pre-defined rules and output signal to point Y, The PLC can immediately drive physical devices or play the expected voice prompts for the user/patient, ultimately achieving the desired extended functionality and user/patient behavior.

The system is designed for different users/patients and can output signal point Y to the PLC based on pre-defined logical control instructions, which can drive physical devices or play the expected voice prompts for the user/patient, ultimately achieving the desired extended limb or verbal expression capabilities for the user/patient. 

The process of transforming DT-HMIS into instruction control commands or reassembling speech is shown in Figure 1. The DT-HMIS has the capability of being user-defined. Users/patients can define their own DT mapping tables for finger gestures and freely decide the mapping ability between user gestures and DT gestures.

As shown in Figure 1, the DT-HMIS allows users/patients to define their own finger gesture mapping model. Users can use real-world finger gestures to point to specific positions in an 8-digit space, and the system will map these real-world gestures to the corresponding DT gestures model based on the user-defined DT mapping gestures model. The system service is constantly supervised by the task command center of the system program, which analyzes the real-time actions of the DT-HMIS sensor and converts them into control commands written to the PLC to control the expansion device or voice player for reorganizing sentences. 

The specific steps are as follows:

Step 1. The user/patient pre-defines a DT mapping model consisting of two-digit number groups and the real meaning represented by each number group.
Step 2. The user points to two positions in a specific 8-digit space using a single finger to generate a set of two-digit number groups.
Step 3. The system automatically refers to the two-digit number group generated in Step 2 and converts it into a corresponding DT gesture command using the user-defined mapping model.
Step 4. The system service continuously monitors the DT gesture commands in the DT mapping model and instantly maps them to write DT signals to the input Xn point of the PLC. When the DT mapping characteristics of the input Xn point of the PLC meet the user-defined computing conditions in the logic unit, the Yn logic point of the PLC is used to control and drive external electronic devices, thereby expanding the limb function of the user/patient.

**Figure 2:**
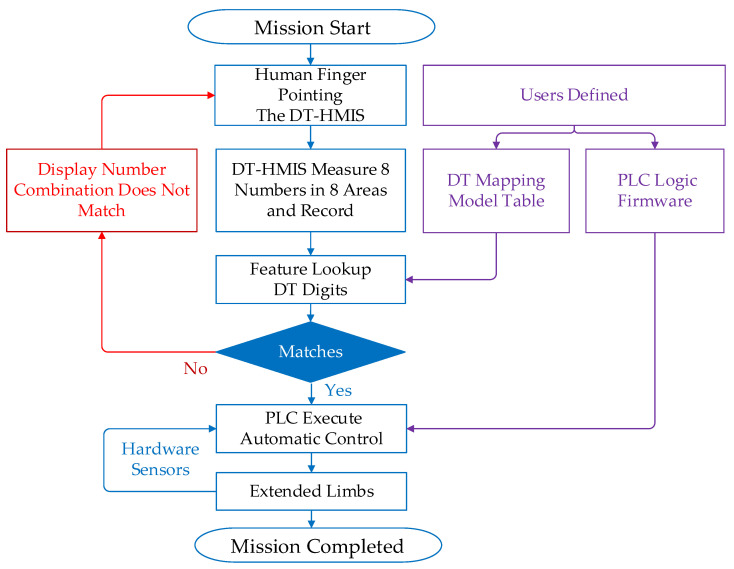
The DT-HMIS service and processing flow chart.

Digital cameras capture dynamic gesture image features, which become DT visual sensors capable of detecting user behavior through algorithms. In this way, the gesture digit models produced by fingers can be monitored through the DT-HMIS service, as shown in Figure 2, reflecting the gesture commands generated by people’s finger combinations. Gesture commands can be converted into effective electronic control and voice feedback signals. In practical applications, the system first initializes the DT image and then uses a digital camera to obtain the virtual image of the finger features in the image—the characteristics of the real-world fingers. In the pre-defined eight-directional digital space, each unique digital command virtual template from the mapping table corresponds to a virtual finger value model in the virtual template when capturing real-world fingers in the camera. This is also the idea of humans in the real world. By combining the finger gesture feature value model with the numerical values in the DT virtual template, changes in the real-world fingers can be determined. Then, by corresponding to the mapping value from the self-mapping table, it can be quantified into a dedicated electronic control signal.

In order to detect moving objects, the first step in image processing is to eliminate background interference. In other words, images and backgrounds must be separated based on image features or background similarity. The theory of image segmentation and removal of dynamic backgrounds is as follows.

### 2.1. Color Space Conversion, Noise Reduction, and Color Stability Improvement

**Figure 3:**
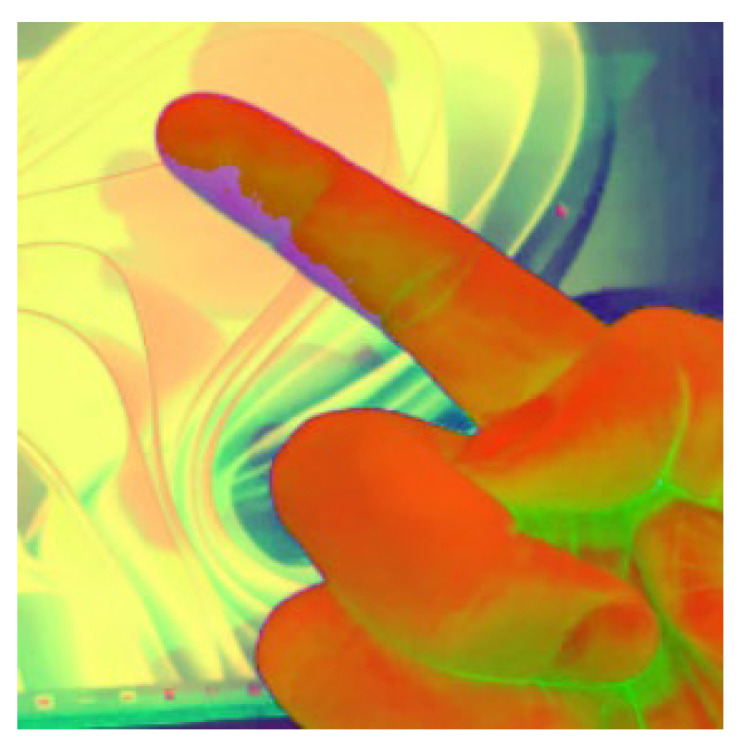
RGB to the HSV processing results.

In image pre-processing, RGB must first be converted to the HSV color space that is less affected by light [19] to facilitate the identification of skin color features of fingers and avoid most color noise. To effectively solve the RGB light source and color interference phenomenon obtained by camera photography, it is necessary to convert it into a more accurate and stable HSV color space, as shown in Equations (1)–(3) [19]. This will generate pseudo-colors, as shown in Figure 3.

(1) $$H=\mathrm{arocos}\left(\frac{\frac{1}{2}\left(2R-G-B\right)}{\sqrt{{\left(R-G\right)}^{2}-\left(R-B\right)\left(G-B\right)}}\right)$$

(2) $$S=\frac{\max\left(R,G,B\right)-\min\left(R,G,B\right)}{\max\left(R,G,B\right)}$$

(3) $$V=\max\left(R,G,B\right)$$

### 2.2. Distinguishing the Problem of Uneven Color Brightness in the Foreground and Background

To address the issue of uneven brightness in foreground colors caused by the uneven distribution of light [20], it utilizes gradient histogram processing to extract features from the processed image. This approach improves finger gesture recognition in natural environments when used in real-time hand pose estimation systems [21], as well as for matching and tracking [22]. The formula for this inference is based on the Equation (1). 

Uneven brightness is a common problem when taking pictures, which can result in some areas appearing too dark or too bright. This can make it difficult to distinguish between the background and finger objects’ light reflection values, and therefore histogram equation of brightness is necessary. In the figure, the display probability of pixel points with gray levels is expressed as Equation (4). The corresponding cumulative distribution function for $P_{x}$ is defined as the cumulative normalized histogram of the image, and the related formula is as follows.

(4) $$Pixel\left(j\right)=P_{x}\left(\mathrm{Gx}=j\right)=\frac{ni}{n},\;0\leq i<z$$

(5) $${\left(cdf_{x}\left(j\right)\right)}^{n}=\overset{j}{\underset{k=0}{\sum }}p_{ixel\left(k\right)}$$

where the discrete grayscale image is represented as x, $P_{x}\left(j\right)$ is the histogram of pixel values in the images, n is the entire pixel value of the images, and z is the 256 grayscale values of the images. $n_{i}$ represents the frequentation of gray value I, normalized to [0,1].

### 2.3. Segmentation of Images Using Otsu’s Algorithm

In DT visual sensors, it is necessary to eliminate the interference of background images to detect objects in visual coordinate motion. In other words, the image must be separated from the background based on the similarity of an image or environmental features in order to distinguish different objects. The encoding of image regions in eight directions is mapped to the PLC. The controller generates electronic signals for driving motor components to produce motion control behavior. Therefore, the Otsu algorithm is used, which is fast and can achieve adaptive threshold control, efficiently obtaining the optimal point. It is less affected by the light source during calculation and is more accurate than ordinary segmentation methods [23]. The details of the algorithm are stated as follows: the distribution of pixel values in the grayscale image (M×N) is $\left[0,1,2,\ldots ,k-1\right]$; the number of pixels with gray level i is n; and the probability distribution of i is $p_{i}$, as shown in the following formula. Any $p_{i}$ is greater than 0, and the sum of all $p_{i}$ is equation to 1.

(6) $$p_{i}=\frac{n_{i}}{MN}$$

If $T\left(t\right)=t,0<t<k-1,$ and the pixels of a gray-scale image are divided into two sets, $C_{0}$ and $C_{1}$, then the scope of the image grayscale of $C_{0}$ is $\left[0,1,2,\ldots ,t\right]$ and the scope of the image grayscale of $C_{1}$ is $\left[t+1,t+2,t+3,\ldots ,k-1\right]$. The probabilities of $C_{0}$ and $C_{1}$ are then $\omega_{0}\left(t\right)$ and $\omega_{1}\left(t\right)$, respectively, as is shown in Equations (7) and (8). Moreover, the average strength values of $C_{0}$ and $C_{1}$ are $m_{0}\left(t\right)$ and $m_{1}\left(t\right)$, respectively, as is shown in Equations (9) and (10):

(7) $$\omega_{0}\left(t\right)=\overset{t}{\underset{i=0}{\sum }}p_{i}$$

(8) $$\omega_{1}\left(t\right)=\overset{k-1}{\underset{i=t+1}{\sum }}p_{i}$$

(9) $$m_{0}\left(t\right)=\frac{1}{\omega_{0}\left(t\right)}\overset{t}{\underset{i=0}{\sum }}ip_{i}$$

(10) $$m_{1}\left(t\right)=\frac{1}{\omega_{1}\left(t\right)}\overset{k-1}{\underset{i=t+1}{\sum }}ip_{i}$$

Therefore, the gray-scale mean of the images is expressed as follows.

(11) $$m=\omega_{0}m_{0}+\omega_{1}m_{1}$$

Hence, the weighted sum of the variables of $C_{0}$ and $C_{1}$ can be obtained from Equation (12).

(12) $$\sigma^{2}\left(t\right)=\omega_{0}{\left(m_{0}-m\right)}^{2}+\omega_{1}{\left(m_{1}-m\right)}^{2}=\omega_{0}\omega_{1}{\left(m_{0}-m_{1}\right)}^{2}$$

**Figure 4:**
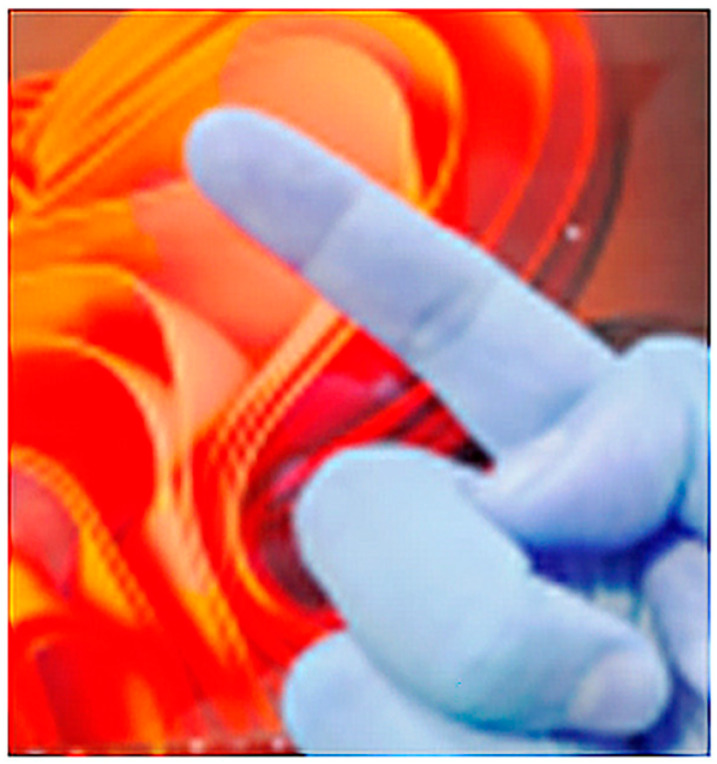
Realize gestures and background segmentation.

Now change the t value in the scope of the gray-scale value $\left[0,1,2,\ldots ,k-1\right]$. When $\sigma^{2}\left(t\right)$ is at its maximum, the t value is the best segmentation threshold. Image segmentation can be easily achieved with this method, as shown in Figure 4.

### 2.4. Derivation of Method for Extracting Object Foreground and Background Differences

The derivation method for extracting the foreground and background differences of an object is described as follows, based on re-executing the gesture search query [24]. In a linear equation of continuous images, the background’s variation is minimal, so finger positioning is divided into two more manageable problems [25], which makes the pixel value differences between the foreground object and background image more significant.

**Figure 5:**
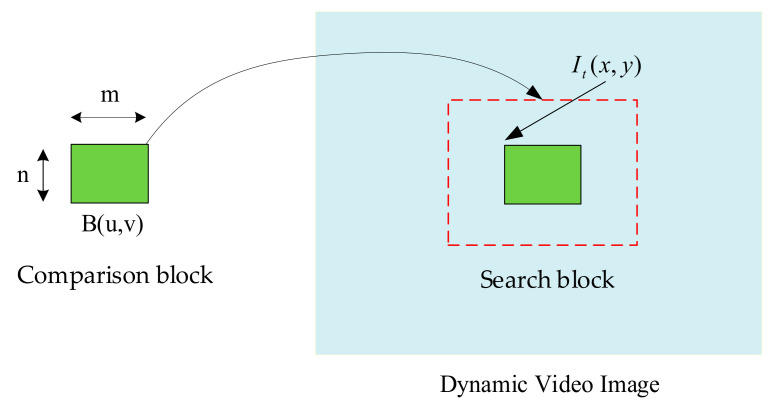
Improved region-based comparative search method.

To achieve the capability of discriminating dynamic gestures or action images [26] for interactive virtual interaction [27], the contrast blocks created are pre-contrasted for each captured dynamic image and thus unrelated to the previous implementation. $B\left(u,v\right)$ represents the image in the comparison window that will be captured in the picture. The search block portion is calculated based on the size of the comparison block. When fingers are bent, and their heights are unsuitable or coordinated with adjacent or stretched fingers, accuracy will be affected [27,28]. Therefore, a window is used to frame the continuous and relatively large skin color to represent the fingertip for simulating mouse pointing functionality [29]. In Equation (13), it indicates that the mean square error (MSE) compares the differences between two dynamic images. Among them, it represents the number of pixels of the window size to be compared, and x and y are coordinate positions relative to the window size, representing dynamic images of time t and time t-1, respectively. As shown in Figure 5, these two images are obtained by comparison, allowing you to know the motion of a moving object. At this time, when the MSE value is the smallest, the center point position measured by the two images is the distance that the relative object moves in the image. If this value is small, the image is more similar [29]. If the value is too large, it is not the search target. The comparison block moves point by point until the target is found.

(13) $$MSE(x,y)=\frac{1}{m\times n}\sum_{i=1}^{i=m}\sum_{j=1}^{j=n}{\left(B_{}(u+i,v+j)-I_{t}(x+i,y+j)\right)}^{2}$$

Hence, the image information of the background can be stored, and the obtained dynamic images are subtracted from the background to remove the background [29] by using Equation (13). Here, $I_{t}\left(x,y\right)$ refers to the grayscale value obtained at the coordinate position $\left(x,y\right)$ in the image at time t; $Bg\left(x,y\right)$ indicates the grayscale value of the background image obtained in advance; and $In_{t}\left(x,y\right)$ is the grayscale value after the calculation. The absolute value can be calculated by Equation (14). Because we want to calculate the pixel value difference between the current image $I_{t}\left(x,y\right)$ and the background image Bg, and this difference value can be positive or negative. Using absolute value can unify the difference value into a positive value, making it easier to compare the different sizes between different pixels for subsection image processing and analysis. If the absolute value is not used, the sign in the calculation result may affect our interpretation and processing of the difference value, leading to errors.

(14) $$In_{t}\left(x,y\right)=|I_{t}\left(x,y\right)-Bg\left(x,y\right)|$$

In an actual environment, the background would change according to the change in light, which makes it impossible to remove all the background. This problem can be improved by the real-time renewal of the background image. Here, $Bg_{t+1}\left(x,y\right)$ is the background image after the renewal, and $\alpha_{t}\left(x,y\right)$ is the offset of a variation. The background images can be evaluated based on the following equation.

(15) $$Bg_{t+1}\left(x,y\right)=\alpha_{t}\left(x,y\right)\cdot In_{t}\left(x,y\right)+\left(1-\alpha_{t}\left(x,y\right)\right)\cdot Bg_{t}\left(x,y\right)$$

Under the influence of lighting, although background subtraction can quickly remove the background, it is difficult to generate effective threshold selection in dynamic operating environments, resulting in poor performance over the long term. Moreover, in some cases, the detected object may be destroyed. Therefore, dynamic object segmentation can be achieved by combining the Otsu algorithm and skin color detection.

**Figure 6:**
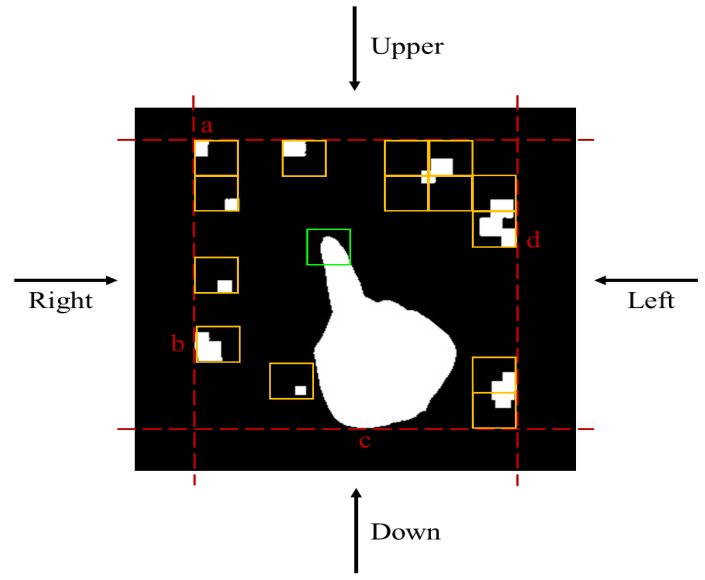
The variation scope of the moving objects.

In Equation (15), we subtract the image obtained from time t−1, from the image obtained from time t and use the Otsu algorithm to obtain a threshold [29]. This helps separate pixel points in areas with significant changes from those with slight changes. Then we equation search the top, bottom, left, and right parts of the image, define the four boundary points of each area, and determine the range of object movement. As shown in Figure 6, the generated black-and-white image is the binary image obtained using this method. However, these images may contain unwanted variation points caused by external factors such as environment or lighting. To solve this problem, we scan the image from the four edges to the center to detect the boundary points of each area, marked as a, b, c, and d in the image. This allows us to define different ranges of motion for the moving object.

In Figure 6, the yellow box represents the finger window, and the system estimates whether the number of skin-colored pixels in the yellow window accounts for the majority of the above area. If the distribution of pixels in the yellow window is too scattered, the system can filter it out and continue to search for the next qualifying area. Once adjacent skin areas that meet the criteria are found, the system marks it with a green box to indicate the target range of the finger has been found. The search will not continue after this point. 

**Figure 7:**
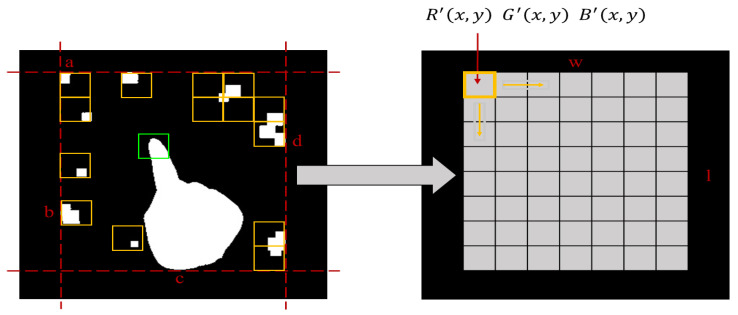
The right diagram shows that a variation area is segmented into small checks.

After defining the region of change, detect the skin color within it to reduce image search time and computational workload. Skin color detection is performed on the color image obtained at time t in the changing region. The first step is to segment the larger defined region and then average the R, G, and B values of the resulting smaller segmented regions to obtain a new pixel value of smaller size. This method is used to avoid signal interference in skin color detection and to reduce point-by-point comparison computations. As shown in Figure 7, since the fingers are relatively long, they need to be segmented into a total of 56 grids of 7 × 8 for processing. The right figure shows the division of the change region (w×l) into small checkboxes (m×n), and each check is calculated by using Equations (15) and (16). The starting coordinates of each check box are represented by (x,y), r(x,y), g(x,y), and b(x,y) are the original pixel values of the point, and R′(x,y), G′(x,y), and B′(x,y) are the newly calculated RGB values. Finally, the original pixel value is replaced by the new pixel value.

(16) $$\left\{\begin{array}{c} R^{\prime }\left(x,y\right)=\frac{1}{m\times n}\overset{i=x+m}{\underset{i=x}{\sum }}\overset{j=y+n}{\underset{j=y}{\sum }}r\left(x,y\right)\\ G^{\prime }\left(x,y\right)=\frac{1}{m\times n}\overset{i=x+m}{\underset{i=x}{\sum }}\overset{j=y+n}{\underset{j=y}{\sum }}g\left(x,y\right)\\ B^{\prime }\left(x,y\right)=\frac{1}{m\times n}\overset{i=x+m}{\underset{i=x}{\sum }}\overset{j=y+n}{\underset{j=y}{\sum }}b\left(x,y\right) \end{array}\right.$$

Each RGB value of a smaller region is converted into the HSV color space, and then the resulting values are evaluated to determine if they are within the skin color range. As recognizing only the hand is not sufficient for accurate control of coordinates, this study focuses on tracking finger features. In order to do this, a window size is set that is the same size as the finger. The skin color portions detected in the dynamic area are marked by pre-processing the image. Then, the window is scanned from top to bottom and from left to right to search for skin color pixels on the upper side of the window. The pixel coordinates are then identified as the center point, and a range is defined around that center point that is consistent with the finger window. Finally, the pixels in the finger window are counted to see if they match the size of the finger. Inconsistent points are considered to be interference points caused by similar skin-like environmental changes.

If the first search fails, a second search is performed until the condition is met. Then, the area is scanned to count the skin color pixels within the window. The distribution of interference points is scattered, so if interference is detected, there are few skin color values in the window. In this case, the end is removed, and another search is performed in the scanning direction until the target point is found.

In order to adapt to people with disabilities, this study solves the position identification problem that may occur during user operation. The system represents the position of the fingertip as (x,y), where M and N respectively, represent the pixels of the length and width of the image. In addition, weight parameters w1, w2, w3, and w4 are used to reduce the influence of different lighting angles, distances, and light background variations, making the system more flexible. The approach is divided into nine rectangular grids, and the code *ft*(x,y) is set to eight directions.

(17) $$ft(x,y)=\left\{ \begin{array}{c} 1,\mathrm{if}\left[x<\left(\frac{N}{3}+w_{1}\right)\right]\wedge \left[\left(\frac{M}{3}+w_{2}\right)<y<\left(\frac{2M}{3}+w_{3}\right)\right]\\ 2,\mathrm{if}\left[x>\left(\frac{2N}{3}+w_{4}\right)\right]\wedge \left[\left(\frac{M}{3}+w_{2}\right)<y<\left(\frac{2M}{3}+w_{3}\right)\right]\\ 3,\mathrm{if}\left[\left(\frac{N}{3}+w_{1}\right)<x<\left(\frac{2N}{3}+w_{4}\right)\right]\wedge \left[y<\left(\frac{M}{3}+w_{2}\right)\right]\\ 4,\mathrm{if}\left[\left(\frac{N}{3}+w_{1}\right)<x<\left(\frac{2N}{3}+w_{4}\right)\right]\wedge \left[y>\left(\frac{2M}{3}+w_{3}\right)\right]\\ 5,\mathrm{if}\left[x<\left(\frac{N}{3}+w_{1}\right)\right]\wedge \left[y<\left(\frac{M}{3}+w_{2}\right)\right]\\ 6,\mathrm{if}\left[x<\left(\frac{N}{3}+w_{1}\right)\right]\wedge \left[y>\left(\frac{2M}{3}+w_{3}\right)\right]\\ 7,\mathrm{if}\left[x>\left(\frac{2N}{3}+w_{4}\right)\right]\wedge \left[y<\left(\frac{M}{3}+w_{2}\right)\right]\\ 8,\mathrm{if}\left[x>\left(\frac{2N}{3}+w_{4}\right)\right]\wedge \left[y>\left(\frac{2M}{3}+w_{3}\right)\right] \end{array}\right.$$

The proposed system is a real-time operation, and many interference signals can affect the recognition results. In this system, a register is used to store the recognition results. When a finger gesture remains still for more than a predetermined number of seconds, it is considered a recognition result and recorded for coding. A temporary code is set as C_0_, a stay duration code is set as C_t_, and the initial values of C_0_ and C_t_ are both 0. Here, C_1_ and C_2_ are the numeric codes of the output function, and their initial values are also set as 0. The constant limits of Equation (17) have been determined by actual testing and adjustment according to background complexity. After obtaining the recognition results, Equation (18) is used for judgment to obtain the numeric codes.

(18) $$\left\{\begin{array}{c} if\;C_{t}=1\;\wedge C_{0}=0,\;Let\;C_{0}=ft\left(x,y\right)\;\wedge C_{t}=0\\ \begin{array}{c} if\;C_{t}=1\wedge C_{0}\neq 0\;\wedge \;C_{1}=0,\;\;Let\;C_{1}=ft\left(x,y\right)\\ \wedge C_{t}=0 \end{array}\\ if\;C_{1}\neq 0\wedge C_{2}\neq 0,output\;function=\left(C_{1},C_{2}\right)\\ \wedge \;\;Let\;\;C_{1}=0\wedge C_{2}=0\\ if\;ft\left(x,y\right)=ft+1\left(x,y\right)=\ldots ft+n\left(x,y\right),\;\;\;\;Let\;C_{t}=1\; \end{array}\right.$$

where $ft+1\left(x,y\right)\;,\;ft+2\left(x,y\right)$… $ft+n\left(x,y\right)$ are the numeric codes at time t, t + 1, t + 2…t + n.

### 2.5. Reassembling and Generating Speech Sentences Using DT Mapping Reference Model and Method

**Figure 8:**
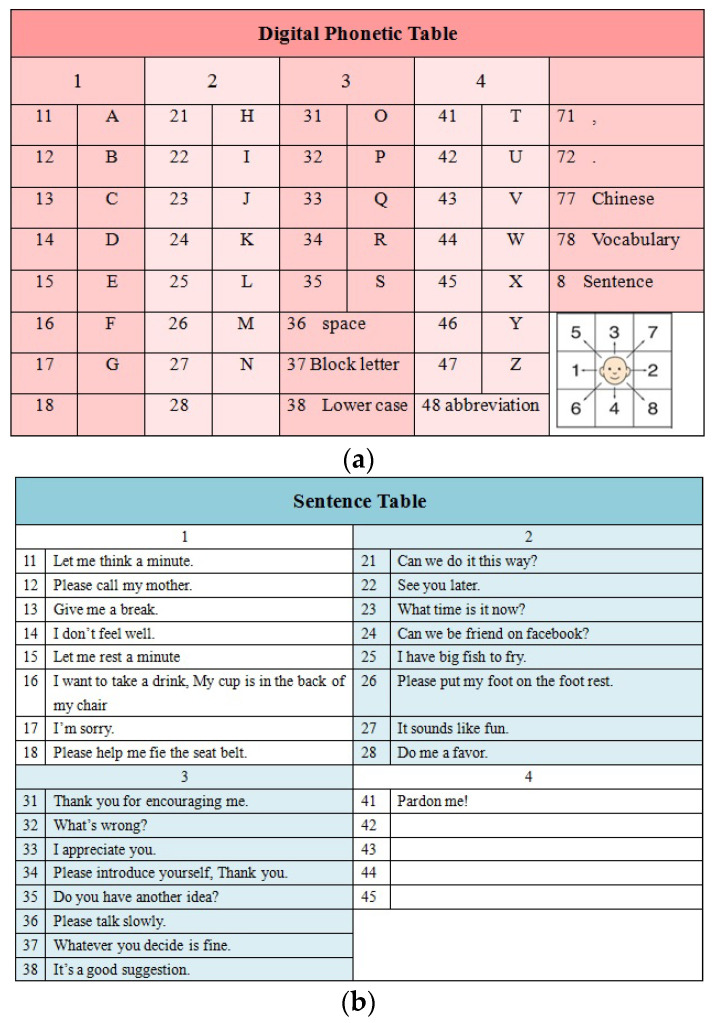
The numeric DT-MHIS phonetic system. (**a**) numeric English phonemicized table and (**b**) frequentation-used sentences.

These numerical combinations form speech correspondences, which are then used to construct sentences. The numerical HMI speech system has strong generality and is used for the digital English table for speech synthesis [29], as shown in Figure 8. By systematically observing the numerical twin human-machine interface sensor mappings from gestures, signals are mapped and used to trigger appropriate sounds, as shown in Table 1.

### 2.6. Combining Finger Gesture Values into Control Commands and then Mapping the Control Signals to the Method of Extending the Limbs

In this study, X0, X1, and X2 are used as finger gesture signals for the input points of the PLC controller, and Y0, Y1, and Y2 are used as control output signals for the PLC output points. When the user slides their finger to the left or right, the system can detect two digits from the continuous sliding and combine them into a command code for further processing. The proposed system uses the commands as shown in Table 2 and analyzes the combination of these values to determine the input and output points of the PLC.

The finger gesture signals of real-world people are accessed through a continuous processing method and TCP/IP protocol network to access the logical contacts corresponding to X0, X1, and X2 in the PLC. Y0, Y1, and Y2 of the PLC are used as logical contacts for control output signals. When the user slides their finger to the left or right, the system can detect multiple digits from the continuous sliding. For example, it may consist of 2 to 10 values to form one command (namely, two digits are used to form one command) and combine them into a command code for further processing. The proposed system uses the commands to analyze the combination of these values to determine the input and output points of the PLC, as shown in Table 2.

For example, when the user slides their finger to form the number 45, it corresponds to the X0 input point in the PLC. After receiving the X0 signal, the bed will rise after a delay of 1 s. When the gesture is removed, the register will reset to 0 after a delay of 0.5 s, and Y0 will be turned off to prevent the bed from rising again. This design implements the physical logic of point control, where power is only supplied when needed.

**Figure 9:**
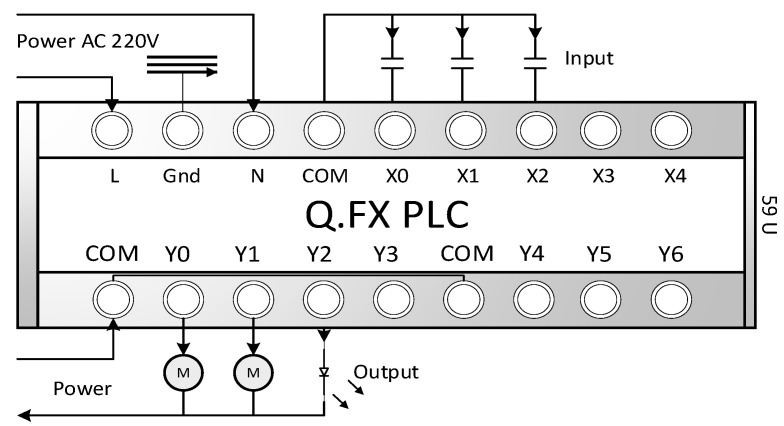
The PLC corresponds to the X~X4 contacts, where X is the input contact, Y is the output contact, M is the external motor, and COM is the common parallel point. The L and N of the power supply represent the live and neutral wires, respectively. The Q and FX models are PLCs introduced by the MITSUBISHI Company. This PLC, designed for this system, allows the control logic program to be updated through the users such as the DT mapping model in DT-HMIS of this system, without being limited by the developer. Users/patients can also extend peripheral device control on their own.

Similarly, when the user forms the number 46 with a gesture, it corresponds to X1. After receiving the X1 signal, the bed will descend after a delay of 1 s. When the gesture is removed, the register will reset to 0 after a delay of 0.5 s, and Y1 will be turned off to prevent the bed from dropping again. This design also implements the physical logic of point control. Figure 9 shows the PLC used for motion control and warning light rules, while Table 2 defines the PLC used for motor control. A self-holding logic circuit was designed to keep the warning light on without flashing. When the user slides their finger to form the number 47, it maps to X2. When X2 is triggered once, the delay of 0.5 s is temporarily stored and held as 1, entering a self-holding trigger state. When the gesture is slid again to form the number 47, it maps to X2 again, and the delay of 0.5 s is temporarily stored and held as 0, ending the continuous trigger state. Therefore, when the user moves their fingers in front of the camera, the system can detect the gesture, generate command codes through the combination of these actions, and determine the input and output status of the PLC through the command code to control the operation of the bed.

**Figure 10:**
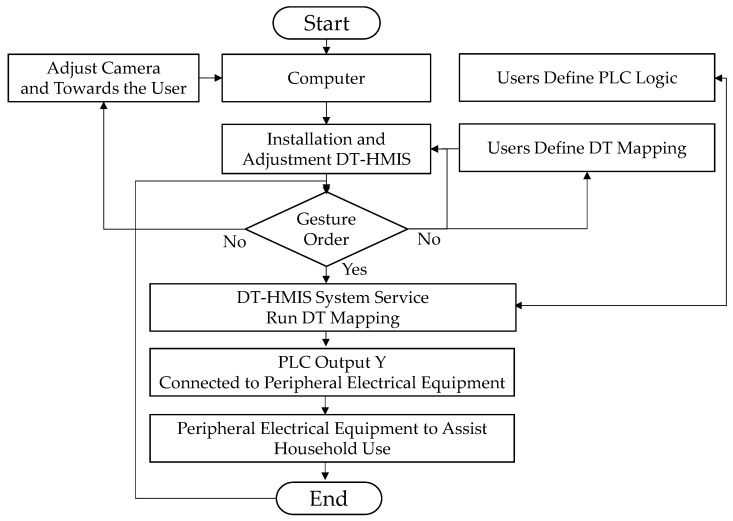
The flow chart of an experimental system.

A self-holding logic circuit was designed for the lamps to maintain a stable brightness and avoid the flicker effect. When the user’s gesture forms the number 47, it maps to the X2 input point. When the X2 contact is triggered, the delay of 0.5 s is temporarily stored and held as 1, entering a self-holding trigger state. When the gesture is slid again to form the number 47, it is activated again and mapped to the X2 input point, and the delay of 0.5 s evolves to 0, and the continuous trigger state also ends. At this time, the PLC’s Y2 output is designed as an odd function output; that is, it is only either 1 or 0. In summary, when the user slides their gesture to the number 47, the light signal will remain on, and when the user slides their gesture again to form the number 47, the light signal will change state and turn off after a delay of 0.5 s. Figure 10 reveals the flow chart of the proposed system operation. Table 3 indicates the equipment and development environment.

## 3. Experimental Results and Discussion

**Figure 11:**
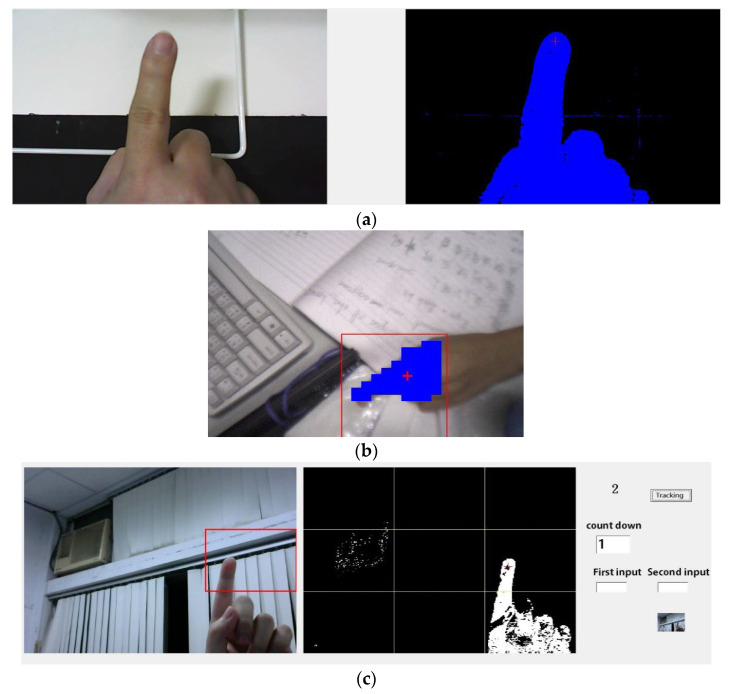
Real-world human finger gesture detection in different operating environments. (**a**) Construction of finger DT model. (**b**) Measurement of real-world fingers by virtual finger models. (**c**) Spatial indications of human finger gestures in the real world.

**Figure 12:**
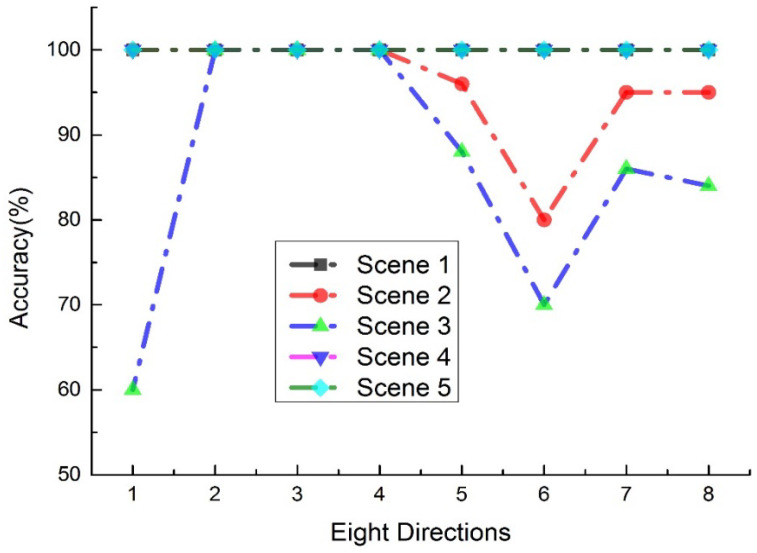
The recognition accuracy of the eight directions in the different backgrounds.

**Figure 13:**
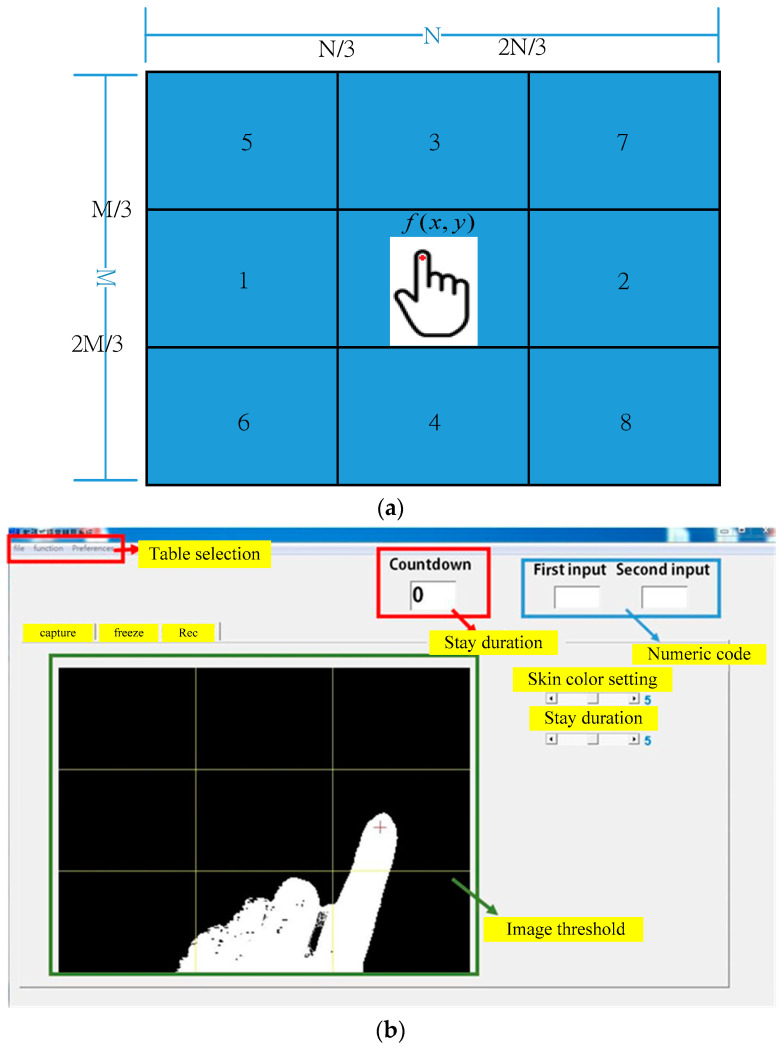
The numeric DT-HMIS using finger gesture recognition (**a**) eight region (**b**) screen of the program.

The experiment was conducted in a typical indoor environment with a total of 22 users operating the system. The experiment did not involve any invasive or therapeutic behavior, only a simple machine vision camera system and peripheral controller to assist patients without physical contact. Five scenes were created, with backlight or interference testing algorithms designed to evaluate the system’s performance under different backgrounds (see Figure 11). The subjects moved in eight different directions five times, and the test results and accuracy were recorded and calculated. The recognition accuracy in eight different directions under different backgrounds is shown in Figure 12. The experiment was repeated 50 times, and the overall average recognition accuracy was 96% (averaged over multiple trials). The digit-human machine interface using gesture recognition is shown in Figure 13.

**Figure 14:**
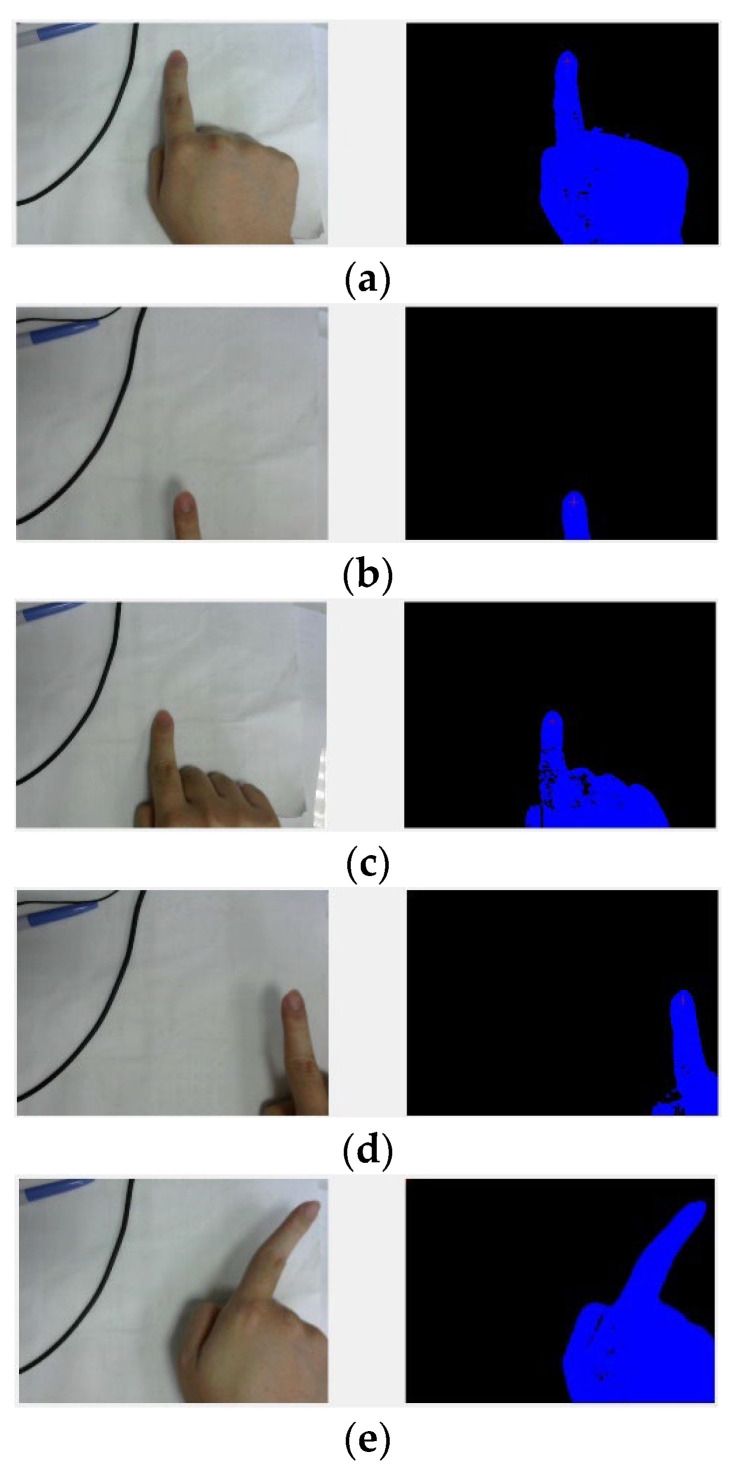

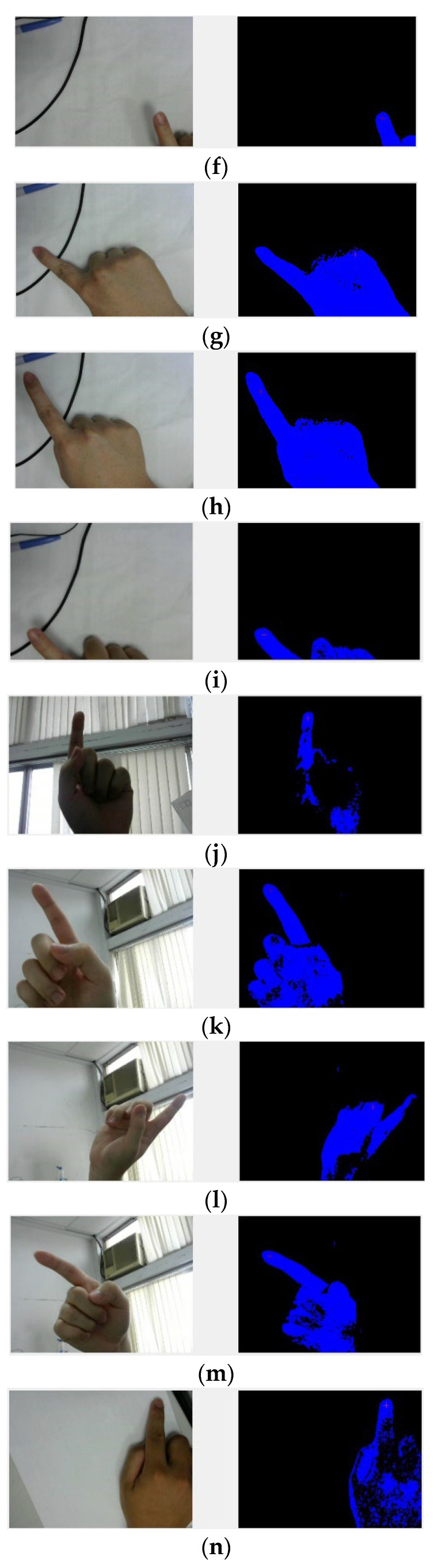
Different finger gesture command parameter identification in various situations: (**a**) Up, (**b**) Down, (**c**) Middle, (**d**) Right, (**e**) Upper right, (**f**) Lower right, (**g**) Left, (**h**) Upper left, (**i**) Lower left, (**j**) Up, (**k**) Upper left, (**l**) Upper right, (**m**) Left, (**n**) Upper right.

This study utilized a camera and software to develop a real-time DT-HMIS for finger gestures, utilizing color recognition through image processing technology to achieve the goal of tracking hand gestures. Adjusting parameters to determine finger gestures and subsequent actions can be distinguished by analyzing the distribution of colors in the image. In this study, RGB was transformed into the HSV color space to reduce the impact of lighting, and foreground segmentation was used to separate the fingers from the background. Command parameters can be flexibly set according to different situations. By using parameters to determine finger gestures, the system can distinguish subsequent actions by analyzing the color distribution in the image. As shown in Figure 14, command parameters can be flexibly adjusted according to different situations. Due to the optical reflection during photography, darker parts of the finger can be easily mistaken for the background. Automatic adjustment can be added to the foreground and background segmentation to ensure a complete display of gestures and improve recognition. The system was trained to improve recognition accuracy.

**Figure 15:**
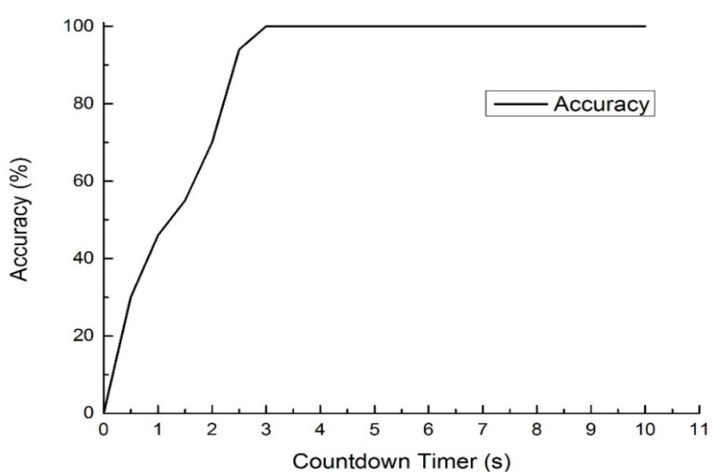
The relationship between the stay duration (countdown timer) and accuracy.

Due to the fact that the hand is a deformable object, if the gestures are too complex, the distribution of characteristic points generated by motion will be very scattered and difficult to analyze. Experimental results show that the system can detect the user’s hand and accurately identify the user. In bright light but complex backgrounds, the recognition accuracy can reach up to 96%. Parameters can be adjusted for different users/patients to obtain higher precision, including sensitivity to the fingers. After adding weight adjustment to Equation (17), this improvement further increased the accuracy to above 99%, demonstrating the system’s stability and usability. Figure 15 shows the relationship between the stay duration (countdown timer) and accuracy.

**Figure 16:**
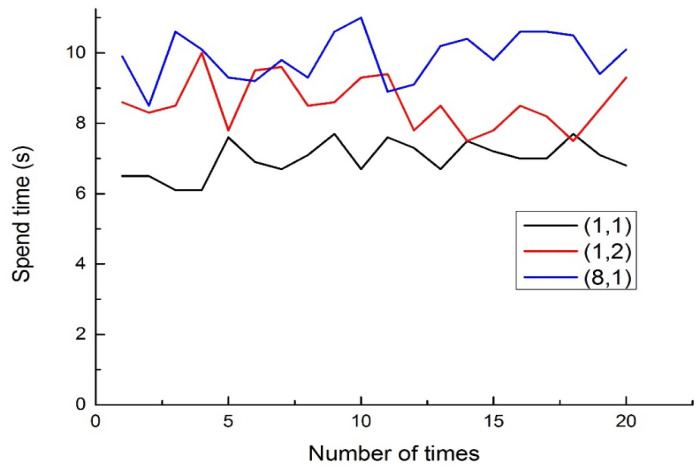
The experimental result of operation time for 20 inputs.

In the real world, there exists a DT-HMIS that allows users/patients to input two-digit commands and virtual spatial positions among eight directions indicated by finger gestures. This is achieved by observing the measurement results of the sensor through system services. People can point their fingers towards these eight directions and associate them with eight numbers, as well as an intermediate position for initialization or an error return. Therefore, in a specific experiment, The DT-HMIS was used to measure the eight numerical values corresponding to the finger gestures pointing towards the eight directions in the real world, which were then combined into a group of mapped reference scripts using two-digit values. Then, by referring to a user mapping table, the DT mapping model script was translated into the corresponding PLC control input point, which achieved the DT command required by people to execute control commands for devices or, as shown in Table 1, to complete the recombination of sentence expressions to convey the user’s meaning through voice. For example, users/patients can use two sets of codes to input commonly used sentences. The test countdown is set to 2 s. The operator can choose the shortest distance (1, 1), the longest distance (8, 1), and the average distance (1, 2) for testing. Before starting the word combination test, the interviewee will have more than 10 min of recording time to familiarize and test. The time required for 20 inputs is shown in Figure 16, and the average time is shown in Table 4.

According to the experimental results above, this system can realize the perception of finger gestures and enable users/patients to input commands to control devices from a dedicated DT mapping table from the users/patients. During the experiment, most people’s memory of the numeric commands improved, and their physical performance also became faster [30]. The self-correction training of finger pointing may have a rehabilitation effect on upper limb movement and should be able to reshape brain areas after brain damage [30]. In short, the system realizes the DT-HMIS system and has many benefits for people, including improving brain memory, stability of limbs, and helping to extend limbs or improve expression ability.

Based on the functional analysis results, the three types of the study can be summarized as follows:

There are two types of devices for human-robot interaction. The first type is wearable and is only applicable to industrial robot arms. However, this type is very expensive and can only operate in sync with the trajectory and angle of the human, making it unsuitable for training and evaluating body response-ability. Moreover, it cannot define customized DT models for users/patients to complete special tasks or interface with other electronic extension devices to achieve extended limb capabilities, nor can it assist with reorganized speech.

The second type is mobile devices with touch capabilities, which are only suitable for evaluating gestures. They cannot customize DT mapping models based on user-defined parameters for age and reflex training, nor can they be used to control external devices to achieve extended limb capabilities.

In contrast, the third type of device proposed in this research is non-contact and does not require wearable equipment, making it more convenient for patients as it does not add weight or require anything to be held in hand. The digital-twin mapping model table and peripheral electronic control logic programs can be defined according to customer needs, and gesture-driven external expansion electronic devices, such as extended limbs and reorganized voice to enhance expression ability, can be customized to meet the specific needs of users/patients. Such a design will significantly improve the convenience and scalability of virtual limb extension.

Table 5 shows that this study identified 22 collaborative features related to biocompatibility. Upon comparison, “Type 3” highlighted the uniqueness and novelty of this study’s characteristics.

In exploring concrete implementation examples, the practicality of virtual limb extension may be evident in the following contexts, for example:

I. Enabling the DT-HMIS collaboration for industrial and domestic applications, such as assisting individuals who cannot touch device switches quickly or directly due to oily or wet hands-on production lines.
II. Used in medical applications [12] for assessing the performance of a patient’s limbs and brain function during awake brain surgery to avoid damage to nerves.
III. Evaluating the recovery status of limbs or brain health before and after surgery.
IV. Assisting patients with mobility impairments to control peripheral electromechanical devices.
V. Helping non-speaking individuals to reorganize vocabulary to form coherent sentences.
VI. Assisting disabled individuals with limb extension training and rehabilitation exercises.
VII. Allowing doctors to use gestures to view X-ray images on a distant screen during surgery.
VIII. Assessing the mental age and maturity of children.

This will significantly improve the convenience and scalability of virtual limb extension.

## 4. Conclusions

This study presents a low-cost, Users-defined Digital-twin human-machine interface sensor (DT-HMIS) that does not require heavy equipment to be worn, using commonly available cameras and algorithms. The proposed DT-HMIS system has many advantages, such as automatic monitoring finger gesture images and being able to control digits with gestures, receiving remote commands from the Users/patients, using reference tables to generate control commands, and ultimately mapping them to a PLC to control mechanical table behavior or external electrical equipment, as well as providing verbal feedback. Additionally, the system can combine gestures and speech, and the speech database used contains commonly used phrases by users/patients and family members. All users/patients can also redefine dedicated gesture commands to the mapping table through the Users/patients, customize and modify corresponding electromechanical integration control, or use them to expand and generate new sentences.

Our research team can design DT-HMIS according to different requirements and control peripheral devices through User/patient updates or provide new speech expressions to meet the needs of different user/patient. This technology can help workers who have only one hand, have limited mobility, cannot quickly approach physical device buttons from a distance, cannot touch switches due to hand contamination, or collaborate in directing self-driving vehicles in noisy environments, in achieving extended limb functionality. Furthermore, the technology can also be applied to dementia patients [12] and potentially be helpful for patients by speeding up thinking and rehabilitation and improving brain memory and motor response capabilities [30].

In future work, there are some improvements needed as only eight (1, 2, 3, 4, 5, 6, 7, 8) digits and boxes are provided for use, omitting the numbers 0 and 9. Since this study only provides instructions composed of two-digit values, it can be improved to allow for instructions composed of multiple digits. Additionally, the central box cannot be used as it is used for initialization or as the starting position when an error occurs. Thus, the digit-mapping model table composed of only eight digits cannot be widely used as a tool to replace telephone dialing services. Furthermore, due to the use of color optical image recognition technology in a restricted environment, there may be an increase in recognition errors in low-light situations. Combining sensors such as ultrasonic or lidar for signal fusion is recommended to improve accuracy in low-light conditions. In terms of user-defined security, the system can define gesture commands to create a personal digital mapping model table for the user to use in the DT-HMIS system. However, to identify vulnerabilities in the system and proactively assess and mitigate threats or exposures to devices and interfaces in IT healthcare systems [31], cross-layer systems should be included in the service design. Additionally, current designs lack hardware encryption such as FPGAs [32], which may pose risks to patient control stability, including “collusion attacks, data leakage and sabotage, malicious code, malware (e.g., ransomware), and man-in-the-middle attacks” [33]. It is recommended to implement version management in the future to track changes in mapping instructions and allow users/patients to verify the authenticity of the digital-twin behavior mapping model table.

## Figures and Tables

**Table 1:** Voice mapping lookup table.

Image Mapping to Device Control Table		
DT Vision Sensing Code	DT Vision Sensing Code	DT Vision Sensing Code
1,1	11.mp3	Let me have a look
1,2	12.mp3	Please call mom for me
8,1	81.mp3	It’s up to you

**Table 2:** Image Mapping to Device Control Table.

DT Vision Sensing Code	Input Logical Point	Electronic Control Signal Output Result
1,1	X0 ON	Y0 Bed Move Up is On
1,2	X1 ON	Y1 Bed Move Down is On
8,1	X2 ON	Y2 Help Lamp is On
8,1	X2 OFF	Y2 Help Lamp is Off

**Table 3:** Equipment and development environment.

Component	Description
Component	Intel Core i9-12900H processor
PC	16GB RAM
RAM	NVIDIA GeForce RTX 3050 Ti with 4GB VRAM
Graphics Card	1080P Resolution and Python & Open CV
Camera and Library	Windows 10
Operating System	MITSUBISHI-Q03UDE, Output Module QY10 DC 24V/2A
Peripheral Equipment	Fan and Bed, and Speaker.
Number of Experimenters	There are 22 people involved in the study, which does not involve any invasive or assigned medical actions and is conducted with voluntary assistance. Therefore, an ethics committee review is not Required

**Table 4:** Timetable for word combination output.

Frequently-Used Command	Output Function	Average Time(s)
Let me have a look and PLC X0	(1,1)	6.99
Please call mom for me and PLC X1	(1,2)	8.58
It’s up to you and PLC X2	(8,1)	9.895

**Table 5:** Comparison of Three Types of DTs’ Capability.

Biocompatibility 22 Items	DT-HMIS Type 3	Wearable Type 2	Touch Type 1
Users-defined DT model	√		
Users-defined control logic	√		
Distant operation	√	√	
Low cost	√		√
Customizable The DT model	√		
Unrestricted light source		√	√
Contact		√	√
Non-contact	√		
Extended limbs	√		
Custom commands	√		
Reorganized word pronunciation	√		
Single finger operation	√		
No need to hold by hand	√	√	
Vitality assessment	√		√
Memory evaluation	√	√	√
Rehabilitation guidance	√	√	√
Age prediction	√	√	√
Training responsiveness	√	√	√
Hands will not get dirty	√		
Not requiring battery power	√		
No risk of electric shock	√		
It won’t hurt	√		

## Data Availability

Not applicable.

## References

[1] Wang H., Lv L., Li X., Li H., Leng J., Zhang Y., Thomson V., Liu G., Wen X., Sun C. (2023). A safety management approach for Industry 5.0’s human-centered manufacturing based on digital-twin. J. Manuf. Syst..

[2] Hultman H., Cedergren S., Wärmefjord K., Söderberg R. (2022). Predicting Geometrical Variation in Fabricated Assemblies Using a Digital-twin Approach Including a Novel Non-Nominal Welding Simulation. Aerospace.

[3] Han X., Lin Z., Clark C., Vucetic B., Lomax S. (2022). AI Based Digital-twin Model for Cattle Caring. Sensors.

[4] Ghandar A., Ahmed A., Zulfiqar S., Hua Z., Hanai M., Theodoropoulos G. (2021). A Decision Support System for Urban Agriculture Using Digital-twin: A Case Study with Aquaponics. IEEE Access.

[5] Sasikumar A., Vairavasundaram S., Kotecha K., Indragandhi V., Ravi L., Selvachandran G., Abraham A. (2023). Blockchain-based trust mechanism for digital-twin empowered Industrial Internet of Things. Future Gener. Comput. Syst..

[6] Davis S.P., Ashayer A., Tabrizi N. Predicting Sex and Age using Swipe-Gesture Data from a Mobile Device. Proceedings of the 2020 IEEE Intl Conf on Parallel & Distributed Processing with Applications, Big Data & Cloud Computing, Sustainable Computing & Communications, Social Computing & Networking (ISPA/BDCloud/SocialCom/SustainCom).

[7] Pulfrey J., Hossain M.S. (2022). Zoom gesture analysis for age-inappropriate internet content filtering. Expert Syst. Appl..

[8] Guarino A., Malandrino D., Zaccagnino R., Capo C., Lettieri N. (2023). Touchscreen gestures as images. A transfer learning approach for soft biometric traits recognition. Expert Syst. Appl..

[9] Nguyen T., Roy A., Memon N. (2019). Kid on the phone! Toward automatic detection of children on mobile devices. Comput. Secur..

[10] Zaccagnino R., Capo C., Guarino A., Lettieri N., Malandrino D. (2021). Techno-regulation and intelligent safeguards. Multimed. Tools Appl..

[11] Gallala A., Kumar A.A., Hichri B., Plapper P. (2022). Digital-twin for Human–Robot Interactions by Means of Industry 4.0 Enabling Technologies. Sensors.

[12] Zhao G.-R., Cheng Y.-F., Feng K.-K., Wang M., Wang Y.-G., Wu Y.-Z., Yin S.-Y. (2022). Clinical Study of Intraoperative Microelectrode Recordings during Awake and Asleep Subthalamic Nucleus Deep Brain Stimulation for Parkinson’s Disease: A Retrospective Cohort Study. Brain Sci..

[13] Yu W., Yamaguchi H., Yokoi H., Maruishi M., Mano Y., Kakazu Y. (2002). EMG automatic switch for FES control for hemiplegics using artificial neural network. Robot. Auton. Syst..

[14] Clark R.A., Pua Y.-H., Fortin K., Ritchie C., Webster K.E., Denehy L., Bryant A.L. (2012). Validity of the Microsoft Kinect for assessment of postural control. Gait Posture.

[15] Hu X., Nenov V. (2004). Multivariate AR modeling of electromyography for the classification of upper arm movements. Clin. Neurophysiol..

[16] Raheja J.L., Chaudhary A., Maheshwari S. (2014). Hand gesture pointing location detection. Optik.

[17] Kılıboz N.Ç., Güdükbay U. (2015). A hand gesture recognition technique for human–computer interaction. J. Vis. Commun. Image Represent..

[18] Lin J., Ding Y. (2013). A temporal hand gesture recognition system based on hog and motion trajectory. Optik.

[19] Mo D.-H., Wu Y.-C., Lin C.-S. (2022). The Dynamic Image Analysis of Retaining Wall Crack Detection and Gap Hazard Evaluation Method with Deep Learning. Appl. Sci..

[20] Knibbe J., Seah S.A., Fraser M. (2015). VideoHandles: Searching through action camera videos by replicating hand gestures. Comput. Graph..

[21] Zhou Y., Jiang G., Lin Y. (2016). A novel finger and hand pose estimation technique for real-time hand gesture recognition. Pattern Recognit..

[22] Suau X., Alcoverro M., López-Méndez A., Ruiz-Hidalgo J., Casas J.R. (2014). Real-time fingertip localization conditioned on hand gesture classification. Image Vis. Comput..

[23] Maqueda A.I., del-Blanco C.R., Jaureguizar F., García N. (2015). Human–computer interaction based on visual hand–gesture recognition using volumetric spatiograms of local binary patterns. Comput. Vis. Image Underst..

[24] D’Orazio T., Marani R., Renò V., Cicirelli G. (2016). Recent trends in gesture recognition: How depth data has improved classical approaches. Image Vis. Comput..

[25] Lee Y.W. (2013). Implementation of an interactive interview system using hand gesture recognition. Neurocomputing.

[26] Rempel D., Camilleri M.J., Lee D.L. (2014). The design of hand gestures for human–computer interaction: Lessons from sign language interpreters. Int. J. Hum.–Comput. Stud..

[27] Lin C.S., Li K.C., Chen C.T., Chang C.C., Chung D.S. (2009). Hand Gesture Recognition in a Leg Sport System. J. Biomed. Eng.-Appl. Basis Commun..

[28] Song Y., Sun Y., Zhang H., Wang F. (2016). Activity testing model for automatic correction of hand pointing. Inf. Process. Lett..

[29] Chang C.M., Lin C.S., Chen W.C., Chen C.T., Hsu Y.L. (2020). Development and Application of a Human-Machine Interface Using Head Control and Flexible Numeric Tables for Severely Disabled. Appl. Sci..

[30] Pisella L., Grea H., Tilikete C., Vighetto A., Desmurget M., Rode G., Boisson D., Rossetti Y. (2000). An ‘automatic pilot’ for the hand in human pos-terior parietal cortex: Toward reinterpreting optic ataxia. Nat. Neu-Rosci..

[31] Markakis E., Nikoloudakis Y., Pallis E., Manso M. Security assessment as a service cross-layered system for the adoption of digital, personalised and trusted healthcare. Proceedings of the IEEE 5th World Forum Internet Things (WF-IoT).

[32] Tao H., Bhuiyan M.Z.A., Abdalla A.N., Hassan M.M., Zain J.M., Hayajneh T. Secured data collection with hardware-based ciphers for IoT-based healthcare. Proceedings of the 2019 IEEE 5th World Forum on Internet of Things (WF-IoT).

[33] Nausheen F., Begum S.H. Healthcare IoT: Benefits, vulnerabilities and solutions. Proceedings of the 2018 2nd International Conference on Inventive Systems and Control (ICISC).

